# Development of Turmeric Oil—Loaded Chitosan/Alginate Nanocapsules for Cytotoxicity Enhancement against Breast Cancer

**DOI:** 10.3390/polym14091835

**Published:** 2022-04-29

**Authors:** Htet Htet Moe San, Khent Primo Alcantara, Bryan Paul I. Bulatao, Waraluck Chaichompoo, Nonthaneth Nalinratana, Apichart Suksamrarn, Opa Vajragupta, Pranee Rojsitthisak, Pornchai Rojsitthisak

**Affiliations:** 1 Pharmaceutical Sciences and Technology Program, Faculty of Pharmaceutical Sciences, Chulalongkorn University, Bangkok 10330, Thailand; htethtetmoesan17.hhms@gmail.com (H.H.M.S.); khentalcantara@gmail.com (K.P.A.); bibulatao@up.edu.ph (B.P.I.B.); 2 Center of Excellence in Natural Products for Ageing and Chronic Diseases, Chulalongkorn University, Bangkok 10330, Thailand; waraluck_kik@hotmail.com (W.C.); nonthaneth.n@pharm.chula.ac.th (N.N.); opa.v@chula.ac.th (O.V.); pranee.l@chula.ac.th (P.R.); 3 Department of Pharmacology and Physiology, Faculty of Pharmaceutical Sciences, Chulalongkorn University, Bangkok 10330, Thailand; 4 Department of Chemistry and Center of Excellence for Innovation in Chemistry, Faculty of Science, Ramkhamhaeng University, Bangkok 10240, Thailand; s_apichart@ru.ac.th; 5 Molecular Probes for Imaging Research Network, Faculty of Pharmaceutical Sciences, Chulalongkorn University, Bangkok 10330, Thailand; 6 Metallurgy and Materials Science Research Institute, Chulalongkorn University, Bangkok 10330, Thailand; 7 Department of Food and Pharmaceutical Chemistry, Faculty of Pharmaceutical Sciences, Chulalongkorn University, Bangkok 10330, Thailand

**Keywords:** *ar*-turmerone, polymeric nanoparticles, anticancer activity, release study, biodegradable polymers

## Abstract

Turmeric oil (TO) exhibits various biological activities with limited therapeutic applications due to its instability, volatility, and poor water solubility. Here, we encapsulated TO in chitosan/alginate nanocapsules (CS/Alg-NCs) using o/w emulsification to enhance its physicochemical characteristics, using poloxamer 407 as a non-ionic surfactant. TO-loaded CS/Alg-NCs (TO-CS/Alg-NCs) were prepared with satisfactory features, encapsulation efficiency, release characteristics, and cytotoxicity against breast cancer cells. The average size of the fabricated TO-CS/Alg-NCs was around 200 nm; their distribution was homogenous, and their shapes were spherical, with smooth surfaces. The TO-CS/Alg-NCs showed a high encapsulation efficiency, of 70%, with a sustained release of TO at approximately 50% after 12 h at pH 7.4 and 5.5. The TO-CS/Alg-NCs demonstrated enhanced cytotoxicity against two breast cancer cells, MDA-MB-231 and MCF-7, compared to the unencapsulated TO, suggesting that CS/Alg-NCs are potential nanocarriers for TO and can serve as prospective candidates for in vivo anticancer activity evaluation.

## 1. Introduction

Breast cancer (BC) is the most common type of cancer to be diagnosed and the primary cause of cancer death among women, according to the Global Cancer Observatory 2020, with an estimated incidence and mortality rate of 24.5% and 15.5%, respectively [1]. Generally, BC can be categorized as estrogen-receptor-positive (ER+) or -negative (ER−). Other types, based on biomarkers such as progesterone receptor (PR) and human epidermal growth factor receptor 2 (HER2), are further sub-categorized as luminal A and B, basal-like, and HER2+ [2,3]. Basal-like BC or triple-negative BC (TNBC) is a unique type due to the absence of the biomarkers ER, PR, and HER2 [4]. The development of an effective treatment strategy for breast cancer remains very complex due to its multifaceted behavior against protein expression. Different types of BC respond differently to treatments, making BA treatment almost intractable. Current therapy for BC involves a multimodal strategy combining surgery, chemotherapy, radiotherapy, adjuvant therapy, and hormonal therapy [5,6,7]. However, long- or short-term use could result in an economic and psychological burden on patients and, worse, a high chance of multidrug resistance and detrimental side effects [8]. Thus, the survival rate of patients with BC is still unsatisfactory. Currently, researchers are leaning toward finding alternative forms of treatment for BC, whether in the form of therapeutics, adjuvant treatments, chemopreventive agents, or effective targeting and delivery systems [9,10]. For many years, phytochemicals have been viewed as novel approaches to the targeting and killing of cancer cells while mitigating the harmful side effects of conventional therapies [8].

Turmeric (*Curcuma longa* L.), which belongs to the family Zingiberaceae, has been used as a traditional home remedy, dye, and food additive in Southeast Asia. One of the major components of turmeric is turmeric oil (TO) which mainly contains *ar*-turmerone. TO has been widely used in pharmaceutical applications due to its broad range of biological activities, particularly its antioxidant [11] and anticancer properties [12]. Previous *ar*-turmerone studies on breast cancer showed the inhibition of enzymatic activity and the expression of matrix metallopeptidase 9 (MMP-9) and cyclooxygenase-2 (COX-2) through the nuclear factor kappa-light-chain-enhancer of activated-B-cells (NF-κB) pathway [13]. Furthermore, this compound was proven to stimulate peripheral blood mononuclear cell (PBMC) proliferation and cytokine production [14]. However, despite numerous reports on promising anti-cancer and immunomodulatory activities, TO possesses various disadvantages, such as instability, volatility, and highly lipophilic properties, limiting its therapeutic applications [15,16].

Recently, nanoparticles (NPs) have been the primary source of interest in therapeutic formulations for amplifying stability, bioavailability, and delivery to the target site [17]. Alginate (Alg) and chitosan (CS) are interesting in pharmaceutical applications due to their non-immunogenicity, biocompatibility, biodegradability, sustained release into the bloodstream or cancerous tissue, and enhanced drug-encapsulating efficiency [18,19]. Various preparation techniques have been developed concerning the production methods of chitosan/alginate nanoparticles (CS/Alg-NPs), including sonication [20], electrostatic gelation [21], the self-assembly of polysaccharides [22], the extrusion of polymer dispersions [23], electrospraying [24], and microfluidic methods [25]. The ionotropic gelation method, based on electrostatic interaction, is one of the most frequently utilized formulation methods [26]. The ionotropic gelation method produces CS/Alg-NPs through pre-gelation and polyelectrolyte complexation phases. While the pre-gelation phase occurs via the ionic cross-linking of divalent cations with Alg, the polyelectrolyte complexation phase occurs via electrostatic interactions between the negatively charged carboxylic acid groups of Alg and the positively charged amino groups of CS [27,28]. CS/Alg-NPs have been reported as useful nanocarriers for the encapsulation of chemotherapeutic compounds. Alternatively, emulsification solvent diffusion/evaporation methods are developed for enhancing the solubility of encapsulated compounds in CS/Alg-NPs through oil/water (o/w) emulsion, using stabilizers such as poloxamers [29]. Kumar et al. [29] and Das et al. [30] revealed the positive effect of poloxamer on the encapsulation efficiency of hydrophobic curcumin in CS/Alg-NPs. Sorasitthiyanukarn et al. [31,32] successfully fabricated CS/Alg nanocarriers using o/w emulsification and ionotropic gelation methods by encapsulating curcumin diethyl diglutarate [31] and curcumin diglutaric acid [32] by improving bioavailability and enhancing anticancer activity.

In 2008, Lertsutthiwong et al. [33] reported an approach to overcome these restrictions by encapsulating TO in a biopolymer network, forming Alg nanocapsules (Alg-NCs). However, the TO-loaded Alg-NCs (TO-Alg-NCs) displayed low stability at room temperature and poor drug loading capacity. To overcome these limitations, CS, a natural polysaccharide consisting of ꞵ-(1→4) glycosidic linked D-glucosamine and *N*-acetyl-D-glucosamine, can be used to coat TO-Alg-NCs to obtain TO-loaded chitosan/alginate nanocapsules (TO-CS/Alg-NCs) for physicochemical property improvement [15,16,34]. However, the information on the biological activities of TO-CS/Alg-NCs is limited, and the CS/Alg-NC system for the encapsulation of TO needs to be developed. Therefore, this study was undertaken to establish TO-CS/Alg-NCs with improved physicochemical characteristics and cytotoxicity against two invasive breast carcinoma cell lines, hormone-dependent MCF-7 (ER+ and PR+) and basal-like MDA-MB-231 (TNBC), both of which are invasive breast carcinoma cells.

## 2. Materials and Methods

Chemicals. TO was purchased from Thai–China Flavours and Fragrances Industry (Nonthaburi, Thailand). The amount of *ar*-turmerone in TO was found to be about 12%. *Ar*-turmerone was provided by the Department of Chemistry and Center of Excellence for Innovation in Chemistry, Faculty of Science, Ramkhamhaeng University (Bangkok, Thailand). CS (MW = 63 kDa, 91.74% DD) was supplied by Marine Bio-Resources (Samut Sakorn, Thailand). Sodium Alg (medium viscosity) and poloxamer 407 were purchased from Sigma-Chemicals (St. Louis, MO, USA). Acetonitrile was purchased from RCI Labscan (Bangkok, Thailand). Absolute ethanol, glacial acetic acid, calcium chloride, and other chemicals were purchased from Carlo Erba reagents (Val de Reuil, France).

Cell Culture. Human breast cancer cells (MCF-7 and MDA-MB-231) and HEK293 were cultured in Dulbecco’s modified Eagle’s medium (DMEM) supplemented with 10% fetal bovine serum and 100 units/mL penicillin/streptomycin (Gibco™ Thermo Fisher Scientific Inc., Waltham, MA, USA) in humidified atmosphere of 5% CO_2_ at 37 °C.

### 2.1. Preparation of TO-CS/Alg-NCs

TO-CS/Alg-NCs were prepared by o/w emulsification of TO in the aqueous solution of Alg followed by ionotropic gelation with calcium chloride and coating with a CS solution using the method previously described by Lertsutthiwong et al. [15], with slight modifications. Briefly, 1% (*v*/*v*) ethanolic TO solution was added dropwise using a syringe pump (NE 100, New Era, Pump System Inc., New York, USA), at a speed of 20 mL/h, into the aqueous Alg solution (20 mL, 0.6 mg/mL) containing poloxamer 407 (0.65% (*w*/*v*)), and continuously stirred at 1000 rpm for 30 min using a magnetic stirrer (Onilab LLC Scientific Inc., MS-H380-Pro, Riverside, CA, USA). The o/w emulsion was then sonicated for 15 min, and calcium chloride solution (4 mL, 0.67 mg/mL) was added and continuously mixed for another 30 min. Subsequently, the CS solution (0.1 mg/mL) was added dropwise into the mixture, followed by continuous mixing for 30 min. The TO-CS/Alg-NC suspension was equilibrated overnight in the dark before characterization.

### 2.2. Physicochemical Characterization

The particle size, polydispersity index (PDI), and zeta potential of obtained TO-CS/Alg-NCs were characterized using a Nano-ZS Zetasizer (Malvern Instruments Ltd., Worcertershire, UK). The particle size and PDI were determined by dynamic light scattering and zeta potential was measured by electrophoretic mobility of the NCs [15]. The morphology of the obtained TO-CS/Alg-NCs was visualized using a transmission electron microscope (JEM-2100, JEOL, Tokyo, Japan). The functional groups and interaction between TO and excipients were analyzed using a Fourier transform infrared spectrometer (FT-IR, PerkinElmer Inc., Boston, MA, USA) at a range of 400–4000 cm^−1^ with a resolution of 2 cm^−1^ and 64 scans per spectra.

The encapsulation efficiency (EE) and loading capacity (LC) were determined via the indirect method and quantified using ultra-high-performance liquid chromatography (UHPLC, Agilent 1290 Infinity II LC System, CA, USA) according to the reported method, with some modifications [15]. The TO-CS/Alg-NC suspension was ultracentrifuged (Ultracentrifuge, Hitachi CP 100NX, Ibaraki, Japan) at 4 °C and 45,000 rpm for 1 h. The settled NCs were lyophilized (Lyophilizer, FreeZone, Labconco, MO, USA) for 24 h, and the unencapsulated TO in the supernatant was determined by UHPLC. Briefly, the collected supernatant was diluted with ethanol and filtered through a 0.45-micrometer syringe filter before injection into an Intersil^®^ ODS-3 column (4.6 mm × 150 mm, i.d., 5 μm) (GL Sciences Inc., Tokyo, Japan) maintained at 33 °C. The mobile phase was a mixture of water and acetonitrile (25:75) in an isocratic elution. The injection volume was set at 20 µL with a 0.5 mL/min flow rate. A diode array detector was used to detect the analyte at a wavelength of 254 nm. The chromatographic analysis data running time was 30 min per sample with standard *ar*-turmerone eluted at a retention time of 12.8 min. The quantity of TO in the NCs was computed as the difference between the total amount of TO initially added into the formulation (TO_formulation_) and the amount of TO present in the supernatant (TO_supernatant_). The EE and LC were evaluated using Equations (1) and (2).

(1) $$\mathrm{EE}\;\left(\%\right)=\frac{\left(\mathrm{TO}\;\mathrm{formulation}\;-\;\mathrm{TO}\;\mathrm{supernatant}\right)}{\mathrm{TO}\;\mathrm{formulation}}\times 100$$

(2) $$\mathrm{LC}\;\left(\%\right)=\frac{\left(\mathrm{TO}\;\mathrm{formulation}\;-\;\mathrm{TO}\;\mathrm{supernatant}\right)}{\mathrm{Dry}\;\mathrm{mass}\;\mathrm{of}\;\mathrm{NCs}}\times 100$$

### 2.3. In Vitro Release and Kinetics Studies

The release study of TO from CS/Alg-NCs was performed using a dialysis diffusion method based on a previous report [35], with modifications. Phosphate-buffered saline (PBS) solution (1 mg/mL potassium dihydrogen phosphate, 2 mg/mL dipotassium hydrogen phosphate, 8.5 mg/mL sodium chloride in deionized water, pH 7.4) and sodium acetate buffer (50 mg/mL sodium acetate in 1% acetic acid, with the pH adjusted with 4.2 g/L sodium hydroxide to pH 5.5) were used, with 40 % (*v*/*v*) ethanol in each medium. A dialysis bag (SnakeSkin™, 10,000 Da MWCO, 33 mm diameter; Thermo Scientific, Illinois, USA) with a molecular weight cut-off at 12,000–14,000 Da (Cellu-Sep^®^ T4, TX, USA) was first soaked in the respective media for 24 h before the experiment. TO-CS/Alg-NC suspension (20 mL) was added into the dialysis bag and sealed with clips on both ends. The dialysis bag was immersed in the release medium (500 mL) and maintained at 37 °C under continuous agitation at 100 rpm. Sampling times were set between 0 and 24 h, wherein 5 mL of medium were withdrawn at specific time points. The withdrawn samples were replaced with an equal volume of fresh medium to maintain sink conditions throughout the experiment. The concentration of TO in the medium was quantified using UHPLC and calculated against the calibration curve. The cumulative TO released (%) was computed based on Equation (3):

(3) $$\mathrm{CR}\;\left(\%\right)=\frac{V_{e}\sum_{i=1}^{n–1}C_{n–1}+{\;V}_{o}C_{n}}{m}\times 100$$

where CR is the cumulative amount of TO released (%), Ve is the sampling volume (5 mL), Vo is the total volume of release medium (500 mL), Cn is the concentration of TO at a particular time point (mg/mL), and m is the total amount of TO in TO-CS/Alg-NCs (mg).

The mechanisms involved in the release of TO from CS/Alg-NCs at pH 5.5 and 7.4 were analyzed using non-linear regression by fitting the release data to different kinetic models using the add-in DDsolver software in Microsoft Excel [36]. The kinetic constant (k) was derived using zero-order kinetics, first-order kinetics, Korsmeyer–Peppas’ power law equation, and Hixson–Crowell’s cube root-of-time equation. The release exponent (*n*) was also determined using Korsmeyer–Peppas’ power law equation. The goodness-of-fit of the release of TO was evaluated by comparing the coefficient of determination (r^2^) of the different models [37]. For the Korsmeyer–Peppas model, the release exponent (*n*) can be categorized into values for *n* < 0.43 corresponding to a spherical matrix and a drug release mechanism with Fickian diffusion, with 0.43 < *n* < 0.85 indicating anomalous transport from spheres and *n* > 0.85 suggesting drug release from spheres by polymer swelling [38].

### 2.4. In Vitro Biological Assay

The cell viability assay was adapted from previous work with modifications [39]. Briefly, the cytotoxicities of the unencapsulated TO, TO-CS/Alg-NCs, and the nanocarrier (CS/Alg-NCs) were evaluated using MDA-MB-231, MCF-7, and HEK293 cell lines. The cells were seeded at a density of 3 × 10^4^ cells per 100 µL into each well of 96-well culture plates and incubated for 24 h. Next, the cells were treated with five serial concentrations of pure TO and TO-CS/Alg-NCs in a serum-free medium and incubated at 37 °C for 24 h. The cytotoxicity of the CS/Alg-NCs was also evaluated at a concentration range of 10 to 60% (*v*/*v*). After 24 h of treatment, the culture medium was removed and 100 µL of an MTT reagent (0.5 mg/mL in serum-free medium) was added to each well and further incubated at 37 °C. After 4 h, the MTT medium was removed, and the insoluble formazan crystals were dissolved by adding dimethyl sulfoxide (DMSO). After complete dissolution, the absorbance was measured at 570 nm using a microplate reader (CLARIOstar, BMG Labtech, Ortenau, Baden-Württemberg, Germany). The percentage of cell viability was calculated using Equation (4):

(4) $$\mathrm{Cell}\;\mathrm{viability}\;\left(\%\right)=\left({\mathrm{OD}}_{\mathrm{Sample}}\right)/\left({\mathrm{OD}}_{\mathrm{Control}}\right)\times 100$$

### 2.5. Statistical Analysis

All experiments were performed in triplicate and data were expressed as mean ± standard deviation (SD). The half-maximal inhibitory concentrations (IC_50_) for TO and TO-CS/Alg-NCs were determined through a non-linear regression-curve-fit analysis. A two-way ANOVA was then used to analyze the cell viability data and IC_50_ values. Tukey’s multiple comparisons test was used as the post hoc test. All statistical analyses were performed using GraphPad^®^ Prism software version 9.3.0 (San Diego, CA, USA), with *p* < 0.05 considered statistically significant.

## 3. Results and Discussion

### 3.1. Preparation and Characterization of TO-CS/Alg-NCs

**Figure 1:**
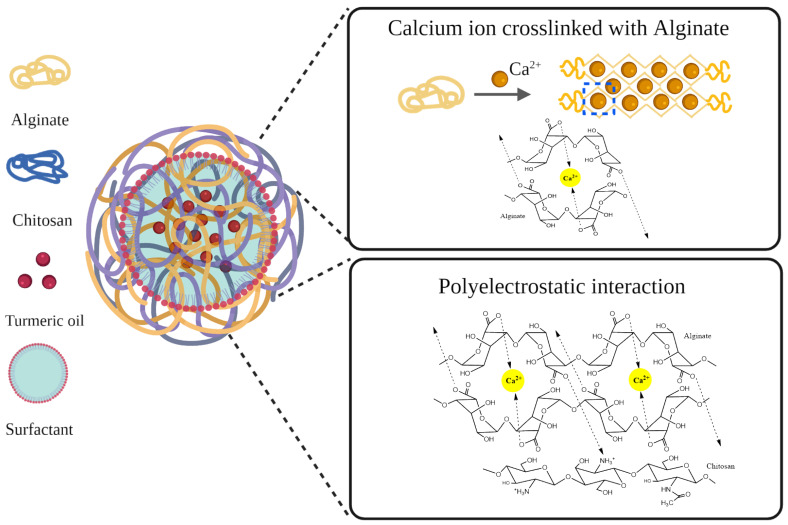
Electrostatic interaction of the anionic Alg with Ca^+^ ions and cationic CS polymer.

**Figure 2:**
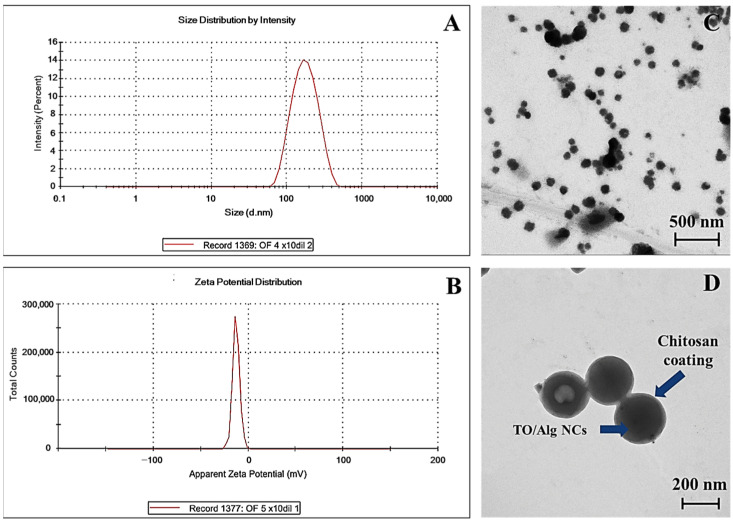
Physical characteristics of TO-CS/Alg-NCs. (**A**): Size distribution by intensity, (**B**): Zeta potential distribution, (**C**,**D**): TEM images at 50,000 and 100,000× magnification, respectively.

The TO-CS/Alg-NCs were prepared by o/w emulsification followed by inotropic gelation based on the previous method, with some modifications [33]. Concerning the procedure of the NC preparation, the NC suspension was used for the subsequent experiments without washing. Although potential impurities, including the organic solvent and surfactant, might have been carried over to the next experiments, these impurities were minimal due to the usage of organic solvent and surfactant at very low concentrations. In addition, the prepared nanosuspension was ultracentrifuged at 45,000 rpm at −4 °C for 1 h to separate the unencapsulated TO and surfactant before lyophilization. The NC preparation was equilibrated overnight to complete the crosslinking process and allow the NCs to form with a uniform size [32]. The o/w emulsification proceeded through the dropwise addition of the ethanolic TO to the aqueous Alg solution containing poloxamer as an emulsifier. Poloxamer is an amphiphilic block copolymer, which consists of poly(ethylene oxide)-poly(propylene oxide)-poly(ethylene oxide) triblock copolymer (PEO-PPO-PEO) [40]. The hydrophobic block in the middle interacted with the TO, while the hydrophilic block on both ends interacted with the aqueous phase, forming micelles. Next, the polymeric Alg micelles with a TO core underwent ionotropic gelation with calcium chloride to form a rigid egg-box structure of oligopolyguluronic sequences, which were presented in the ionic form of the carboxylate group of Alg crosslinking with calcium ion in mildly acidic pH [41]. Following ionotropic gelation, a polyelectrolyte complex between the protonated amino groups of the CS and the ionized carboxylate groups of the Alg was formed. The strength of polyelectrolyte complexation is mainly affected by the pH [42], and, therefore, the carboxylate groups of Alg are ionized at a pH greater than its pKa of 4.4 [43]. However, the amino groups of CS are protonated at pH less than its pKa of 6.5 [44]. In this study, we adjusted the Alg and CS solution to pH 4.9 and 6.0, respectively, to provide sufficient protonated and ionized groups for electrostatic linkages. The magnitude of the electrostatic interaction between the Alg and the Ca^+^ ions and cationic CS polymer could have affected the characteristics of the NCs. High interaction is represented as positive or less negative zeta potential values due to the conservation of the free cationic groups on the surfaces, resulting in the more rigid and compact structure of the NCs (Figure 1) [27]. The results showed an average size of 184.8 ± 14.8 nm and a PDI of 0.192 ± 0.1, indicating the monodispersity of the particles (Figure 2A). The relatively small particle size obtained using poloxamer 407 as a stabilizer was attributed to its higher HLB value (HLB = 18) than Tween 80 (HLB = 15), which was used in a previous study [15]. Surfactants possessing higher HLB values are more suitable for o/w emulsification due to their high water solubility; thus, less aggregation occurs, resulting in more stable and smaller particles [45]. The zeta potential was also determined to predict the stability of the NCs in the aqueous system based on their surface charge. The NCs rendered a negative zeta potential value of about −21.8 ± 1.1 (Figure 2B). Wu et al. [46] suggested that a nanosuspension formulated using poloxamer 407 as a non-ionic surfactant is stable if the zeta potential ranges between −20 and −30 mV. Thus, the use of poloxamer 407 in this study can improve the stability of the NCs.

The morphology of the TO-CS/Alg-NCs was visualized by TEM after diluting the nanosuspension 50× in ultrapure water. The NCs were spherical and had smooth surfaces, with a particle size of approximately 200 nm (Figure 2C,D). It was evident in the image that a thin layer of CS was coated onto the TO-Alg-NCs, as indicated by the arrow shown in (Figure 2D). The EE and LC were determined by the indirect method. The concentration of the unencapsulated *ar*-turmerone in the TO-CS/Alg nanosuspension was determined using the calibration curve of the standard *ar*-turmerone (y = 86.082x + 10.617, R^2^ > 0.9999). The EE and LC of the TO-CS/Alg-NCs using poloxamer 407 were 70.3 ± 1.3% and 3.4 ± 1.3%, respectively. The LC was similar to that in the previous report using Tween 80 as a surfactant [15]. However, the EE of the TO-CS/Alg-NCs using poloxamer 407 (HLB = 18) was higher than that of using Tween 80 (HLB = 15). Ranjith and Wijewardene [47] suggested that a high HLB value renders more water-soluble stabilizers; hence, hydrophobic drugs such as TO can be more stable in an aqueous phase, resulting in high encapsulation efficiency.

**Figure 3:**
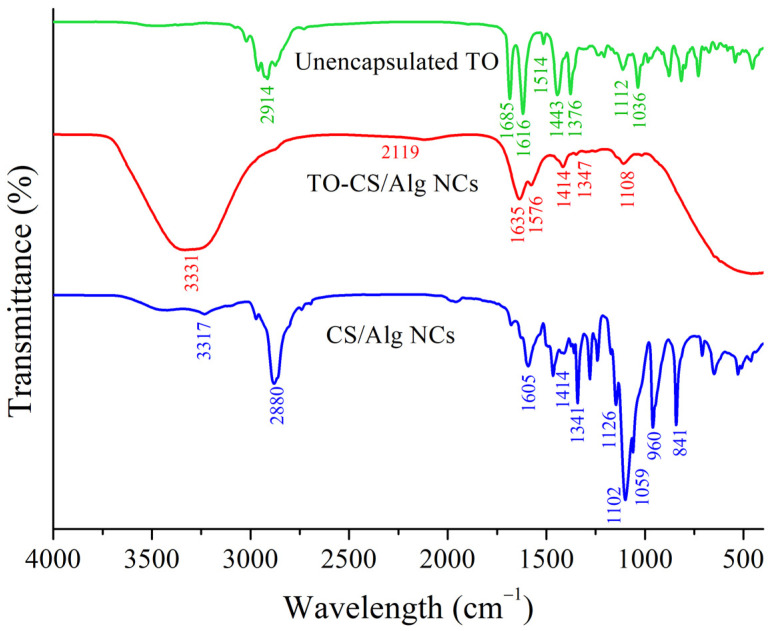
Fourier transform infrared spectra of unencapsulated TO, TO-CS/Alg-NCs, and CS/Alg-NCs.

Further characterization was carried out by FT-IR to investigate possible interactions on the TO-CS/Alg-NCs (Figure 3). In the empty NCs, an IR peak at 1102 cm^−1^ represented –CH–OH in cyclic alcohol and C–O stretching of the CS, while the peak near 1341 cm^−1^ belonged to the C–H bending in poloxamer 407. The peaks at about 1605 cm^−1^ and 1414 cm^−1^ were attributed to the stretching of the –COO- groups in the Alg and the N–H twisting vibration of the essential amine of CS, respectively. These results suggest the interaction between the –COO^-^ of the Alg and the –NH_3_^+^ of the CS to form a strong polyelectrolyte complexation and the encapsulation of the TO in the CS/Alg-NCs [48]. In the spectrum of the TO, a peak at 1685 cm^−1^ could be assigned to the C=O group from the active component, *ar*-turmerone. The bands between 3100 cm^−1^ and 2900 cm^−1^ were attributed to the –OH group or the –NH and aliphatic C–H of the TO [49,50]. Additionally, the TO peaks, 1112, 1618, and 1685 cm^−1^, shifted to 1108, 1576, and 1635 cm^−1^, respectively in the TO-loaded CS/Alg-NCs, indicating the presence of the C=O and CH–OH functional groups, respectively [49]. Moreover, most of the characteristic absorption bands of the TO in the spectrum of the TO-CS/Alg-NCs broadened with the reduction in the intensity, indicating the successful encapsulation of the TO into the CS/Alg-NCs.

### 3.2. In Vitro Release Study of TO

The pH-responsive behavior of the release of the TO from the TO-CS/Alg-NCs was observed in the buffers simulating the blood/normal tissues (pH 7.4) and the acidic endosome environment (pH 5.5) of the cancer cells [51]. Changes in pH changes may be observed as NCs traverse different biological compartments in the human body. For the characterization of the release profile of hydrophobic molecules, the release medium often includes solubility-enhancing agents to better capture the in vivo performance of a drug delivery system [52]. The actual release of the TO from the TO-CS/Alg-NCs was also a function of the mild shear stress due to the constant agitation of the system while incubating at 37 °C. This process mimicked the movement of extracellular fluids around the particles [52]. The NCs inside the dialysis bag contained only the aqueous dispersion of the NCs. The presence of ethanol (40% (*v*/*v*)) in the release buffers, contained in the receiver compartment, facilitated a faster molecular diffusion of the TO throughout the aqueous phase [53]. Thus, it served to maintain a sink condition, preventing the back diffusion of the TO from the receiver to the donor (dialysis bag) compartment [54]. In fact, the use of ethanol in release experiments ranges from 10 to 96%, alone or in combination with water or buffers [35,55,56,57,58,59,60].

**Figure 4:**
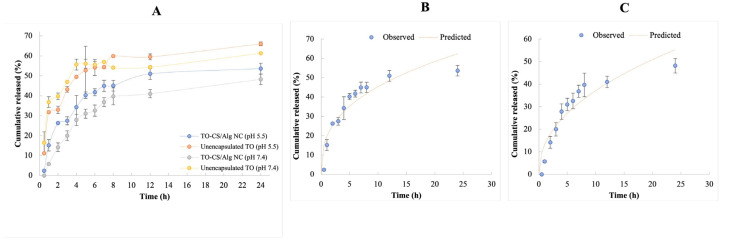
Release study showing (**A**) cumulative release of TO from unencapsulated TO and TO-CS/Alg-NCs in the release media, and nonlinear curve fitting to the experimental data using Korsmeyer–Peppas model in (**B**) pH 5.5 and (**C**) pH 7.4.

Nonetheless, the present study acknowledges some limitations on the conditions of the release experiments that may have had a substantial influence on the quantification of the release of the TO. As is evident in Figure 4A, the slow release profile of the unencapsulated oil in both release media may suggest non-sink conditions, which may have taken the form of the precipitation of the TO inside the dialysis chamber or the affinity of the TO with the dialysis membrane, preventing the unhindered permeation of the TO and its translocation to the receiver compartment [61]. This was evidenced by the apparently large variations in the % TO released in largest number of time points and the absence of a trend showing the immediate and complete release of the TO. However, it can be observed in Figure 4A that the release of the TO from the CS/Alg-NCs provided a biphasic pattern that is typically observed in polymeric NPs. This profile is characterized by a burst release effect followed by a slow release phase [62]. Phase 1 is characterized by the rapid release of TO molecules that are surface-bound or near the water layer. This may be attributed to the diffusion and migration of TO molecules during the fabrication and drying processes, leading to burst release effects as water molecules move to the gel surface, carrying TO molecules via convection and leading to higher TO concentrations at the surface of the carrier. Phase 2, characterized by a slow release, results in a few TO molecules diffusing from the core to maintain a slow and sustained release [63]. This effect can be governed by slow TO diffusion through the polymer matrix or existing pores and is simultaneous with polymer hydrolysis and degradation [62]. Moreover, the diffusion of water would hydrolytically break the bonds of the polyelectrolyte complex [62].

The lower standard deviations in the release of the TO from the TO-CS/Alg-NCs in both release media may imply the efficiency of the CS/Alg-NCs at entrapping, releasing, and dispersing the hydrophobic TO within the dialysis chamber. The efficient release from the NCs could maintain the sink conditions by ensuring that the TO concentration in the receiver compartment was low compared to the TO concentration within the dialysis chamber throughout the entire release study. These reasons are based on a number of assumptions, such as the improved dispersion of the hydrophobic TO due to its encapsulation in the CS/Alg-NCs. In fact, the amount of compound in the receiver compartment should not exceed more than 80% of the total amount of compound used in the experiment [54]. This requirement was achieved, since less than 80% of the TO was translocated to the receiver compartment. This result was important to overcome the effect of the receiver compartment in driving osmosis across the dialysis membrane [64].

In addition, the swelling of polymers and the penetration of fluids into the particles may well take a finite amount of time. This argument may implicate the overall release study and potentially underestimate the release profile of the TO when the release experiment was halted before reaching 100% TO. However, the 24-h dynamic dialysis method to determine the release profile was used in conjunction with the duration of the cell experiment, which was also set to 24 h. With reference to Section 3.3, the slow liberation of the TO from the CS/Alg-NCs may have been responsible for maintaining a therapeutic intracellular concentration of TO, resulting in the reasonable cytotoxicity of the TO in the breast cancer cells. This was the case when CS-NPs encapsulated TO in a previous report, with <30% of the TO released within 24 h in both neutral and acidic media [65]. The same study revealed that extending the release study even for up to 20 days only released the TO by 52%. Our previous study utilized CS/Alg NPs to encapsulate TO, but the in vitro release profile and kinetics were not characterized [65].

Based on the zeta potential of the TO-CS/Alg-NCs (−21.8 mV), it was evident that the negative charge was due to the higher charge density of the carboxyl groups of the Alg, resulting in a higher cross-linking with the CS, a stronger polyelectrolyte membrane, and smaller pores. This was desirable, as an excess of CS results in a positively-charged value for zeta potential, producing increased hypertonicity, which, in turn, increases the osmotic pressure on the polyelectrolyte membrane and leads to a premature bursting effect [37]. A previous study demonstrated that the swelling of the polyelectrolyte complex (PEC) of CS and Alg was higher in acidic than in neutral conditions. At pH 5.5, a stronger PEC membrane is expected, since the CS and Alg are still completely ionized, increasing their counterion charge density [37]. However, the presence of counterions in the buffer solutions results in the charge neutralization of the particles, bringing the apparently negatively charged functional groups of the TO-CS/Alg-NCs close to the isoelectric point [66,67].

Furthermore, the TO release at pH 5.5 was higher than that at pH 7.4. The favored release at pH 5.5 can be explained by the protonation of the NH_2_ groups of CS (up to pH 6.8), which were randomly distributed along with the PEC, resulting in the swelling of CS and promoting the release of the TO through the porous CS/Alg-NCs into the release media [63]. By contrast, the TO from the NCs was slowly released at pH 7.4 because of the decreased solubility and shrinkage of the CS, which prevented the release of the TO from the CS/Alg-NCs [32,37]. At higher pH, the solubility of CS decreases. These observed effects are desirable, since TO should be preferentially released within the acidic intracellular environments of cancer cells [63]. These results demonstrate the pH-responsiveness of the TO-CS/Alg-NCs, which can be regarded as useful in the delivery of compounds due to the differential pH that can be observed in cancerous and normal tissues [68]. Therefore, the CS/Alg-NC carrier is suitable to deliver TO by relying on pH, an internal stimulus that is practical, convenient, and non-invasive in the human body [69].

Comparing the r^2^ among the release kinetic models in Table 1, the highest value was attained for the Korsmeyer–Peppas model. This implies that the Korsmeyer–Peppas’ power-law release kinetics was the best-fit model for describing the release behavior of the TO from the CS/Alg-NCs in both release media (Figure 4B,C). The release exponent (*n*) of the Korsmeyer–Peppas model can be used as a parameter to analyze the configuration of the nanoparticle and the release mechanism involved. Drug release mechanisms from polymeric NPs can be classified into diffusion through water-filled pores, diffusion through the polymer matrix, osmotic pumping, and erosion [62]. It was apparent that the zero-order release profile did not appropriately represent the release of the TO from the CS/Alg-NCs. The values of *n* at pH 5.5 and 7.4 were 0.356 and 0.455, respectively. Since the *n* value at pH 5.5 is less than 0.43, TO-CS/Alg-NCs can correspond to a spherical matrix and a drug release mechanism with Fickian diffusion. In this type of observed behavior, the rate of solvent diffusion is much greater than the process of polymeric chain relaxation, promoting the rapid equilibration of solvent on the surface exposure of the polymeric system. On the other hand, a value between 0.43 and 0.85 can be associated with non-Fickian diffusion, specifically an anomalous transport from the spheres. In this case, the release of TO from the polymeric matrix was governed by the diffusion or swelling of the matrix. Moreover, the velocity of the solvent diffusion and the polymeric relaxation possessed similar magnitudes. These observations may be attributed to the differences in solubility in different media [70]. Additional in vitro release studies would be valuable to assess time points beyond 24 h to better capture more predictive results for in vivo studies.

### 3.3. In Vitro Cytotoxicity Assay

**Figure 5:**
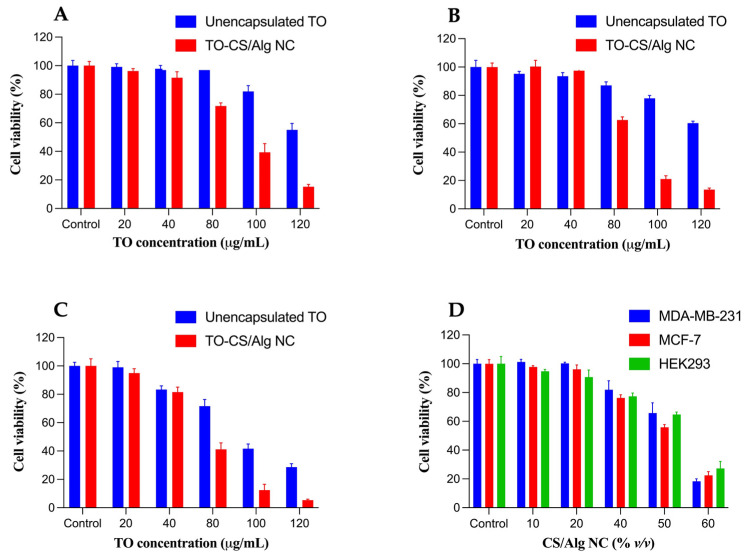
The viability of (**A**) MDA-MB-213, (**B**) MCF-7, and (**C**) HEK293 cells treated with TO and TO-CS/Alg-NCs (equivalent to 20 to 120 µg/mL TO), and (**D**) MDA-MB-231, MCF-7, and HEK293 cells treated with 10 to 60% (*v*/*v*) of CS/Alg-NCs.

The cytotoxic effects of the unencapsulated TO and TO-CS/Alg-NCs (with equivalent TO concentrations ranging from 20 to 120 µg/mL) were evaluated against the MDA-MB-231 and MCF-7 breast cancer cells using the MTT reduction assay (Figure 5A,B). The two breast cancer cell lines were chosen for comparative purposes and to differentiate the responses of the cancer cell lines to the treatments. The MDA-MB-231 and MCF-7 cells are commonly used because they differ in origin, survival, and recurrence rates. The MCF-7 cell line, which originates from human breast adenocarcinoma, is non-metastatic and positive for estrogen receptor (ER+) and progesterone receptor (PR+). The MDA-MB-231 cells belong to the triple-negative breast cancer subtype, which lacks the estrogen receptor (ER), progesterone receptor (PR−), and human epidermal growth factor receptor (HER2−). MDA-MB-231 cells are highly invasive and commonly represent late-stage breast cancer [71,72]. The possible off-target effects of the unencapsulated TO and TO-CS/Alg-NCs were evaluated using HEK293 cells (Figure 5C). The HEK293 is the human embryonic kidney epithelial cell line commonly used to study the toxic effects of nanoparticles [73]. It is an appropriate model as a non-breast-cancer cell line since it demonstrates no tissue-specific gene expression signature, and differentiation markers of several tissues are highly expressed [74].

ANOVA was applied to the cell viability data to determine if there was a significance in the mean difference among the unencapsulated TO and TO-CS/Alg-NCs across the cell lines. The results indicate no significant differences in the cell viability across all the treatments in the cancer cells and HEK293 cell line at 20 µg/mL of TO. Evaluating the safety of the nanocarrier system in a normal cellular environment is an essential tool to ascertain the suitability of the biomaterial for administration to the human body. Therefore, the biocompatibility of the nanocarrier (CS/Alg-NCs) was evaluated using the HEK293 cells (Figure 5D). The present study highlights pronounced toxicity toward HEK293 cells of at least 50% (*v*/*v*) in the CS/Alg-NCs, with the HEK293 cells showing the viability of less than 70%. Figure 5D also shows that up to 40% (*v*/*v*) of the CS/Alg-NCs were considered non-toxic (>70% viability) toward HEK293 and the two breast cancer cell lines, demonstrating the most relevant concentration of the nanocarrier to be considered for subsequent studies. The TO alone did not induce significant cell death in the MDA-MB-231 and MCF-7 cells, even up to 80 µg/mL. The cytotoxic response was augmented when the TO was encapsulated in the CS/Alg-NCs. At 40% (*v*/*v*) of CS/Alg-NCs, equivalent to 80 µg/mL of TO in the TO-CS/Alg-NCs, significant percentages of the MDA-MB-231 (28%) and MCF-7 (40%) were inhibited. Furthermore, both the MDA-MB-231 and the MCF-7 had at least a 60% reduction in cell viability at 100 µg/mL. At 120 µg/mL, at least 80% of the cells were not viable, demonstrating that the CS/Alg-NCs enhanced the toxicity of the TO against the MDA-MB-231 and MCF-7. This demonstrates the TO-concentration-dependent cytotoxicity of the MDA-MB-231 and MCF-7. Overall, the sensitivity of both the MDA-MB-231 and the MCF-7 to the TO-CS/Alg-NCs was prominent between 80 to 120 µg/mL, confirming the cytotoxicity of the TO nanocapsules in different subtypes of breast cancer cells. This implies that the internalization of TO can be enhanced through its encapsulation in CS/Alg-NCs and the endocytosis of TO-CS/Alg-NCs. The factors that may have contributed to the enhanced activity of the TO-CS/Alg-NCs toward the breast cancer cells include their size <200 nm, negative zeta potential, and spherical shape. The negative zeta potential (−21.8 mV) implies that the primary amine groups of the chitosan may have electrostatically interacted with the carboxyl groups of the alginate. On the other hand, this result also indicates that there is an increased association among the hydrophobic acetyl groups of chitosan, promoting stronger hydrophobic interactions with cancer cell membranes [75]. Moreover, the TO-CS/Alg-NCs, which have a negative surface charge, may act as proton sponges, increasing the osmotic pressure within the endosome to release the TO within the cytosol [76]. The significantly higher cytotoxic effect of the TO-CS/Alg-NCs compared to the unencapsulated TO may also be attributed to the sustained release of the TO from the CS/Alg-NCs in the acidic intracellular pH. Concerning the release of the TO from the CS/Alg-NCs, the observed release profile could suggest that a sufficient amount of TO might not have been liberated within the duration of the release study. Nevertheless, the cell viability results imply that the TO encapsulated in the CS/Alg-NCs showed enhanced cytotoxic activity compared to the TO alone, providing evidence that a sufficient concentration of TO was released in the cytosol. The chitosan–alginate polyelectrolyte (PEC) complex was needed to protect the TO and release most of it intracellularly. The PEC may be responsible for the higher mechanical strength of the nanocarrier against possible degradation from chitosanases and lysozymes than either polymer alone [76]. Another possible mechanism underlying the enhanced cytotoxicity of the TO-CS/Alg-NCs includes the presence of the CD44 receptor, a transmembrane glycoprotein, in the MDA-MB-231 and the MCF-7. Although this mechanism may be more relevant for hyaluronic acid, the natural ligand of CD44, its structural similarity to chitosan through the functional group *N*-acetyl glucosamine could have led to the binding and endocytosis of the nanocapsules. Studies have shown that the level of CD44 in MDA-MB-231 was four times as high as that in MCF-7 cells [73,77]. Considering this plausible mechanism, this study partly demonstrates the prominent differential susceptibility of the luminal subtype MCF-7 and the basal subtype MDA-MB-231 to cytotoxic compounds, confirming the documented resistance of MDA-MB-231 to most of the chemotherapeutic drugs, including TO, in the present study [72].

The IC_50_ values of the TO and TO-CS/Alg-NCs’ activity against the MDA-MB-231, MCF-7, and HEK293 cells are presented in Table 2. The IC_50_ values of the TO-CS/Alg-NCs differed significantly from the IC_50_ values of the unencapsulated TO against the MDA-MB-231 and MCF-7, indicating that the TO-CS/Alg-NCs had significantly higher cytotoxicity against the MDA-MB-231 and MCF-7 than the equivalent dose of the unencapsulated TO. The cytotoxic effects of the TO-CS/Alg-NCs observed in both breast cancer and normal cells demonstrate the non-selectivity of the nanocarriers.

The potential off-target effects of the TO-CS/Alg-NCs (at an equivalent concentration of at least 80 µg/mL TO) were shown by the reduced viability of HEK293 cells of 41%. Since the nanocarrier CS/Alg-NCs resulted in a >70% viability for HEK293 cells at the equivalent concentration, the significant off-target effects imply that the reduced viability resulted from the encapsulation of the TO in the CS/Alg-NCs. The cell death of HEK293 cells may have come from the wide variety of phytochemicals present in the TO [78]. Based on the cell viability findings of the present study, a possible strategy to overcome this limitation is to optimize the concentration of TO in CS/Alg-NCs to achieve an optimum therapeutic efficacy while reducing the toxic side effects. The cytotoxic effects may also be attributed to the components of the nanocarrier system itself. Nanoparticle-induced apoptosis in both normal and cancer cells can occur via ROS generation triggering the activation of caspase 9, consequently inducing the intrinsic apoptosis pathway through the mitochondria [79]. Therefore, another strategy to overcome these unwanted findings is to conjugate a ligand with CS. The selective toxicity toward breast cancer cells can be increased by targeting the folate receptors, which are highly expressed in breast cancer cells. The nanocarrier can result in even higher cytotoxicity toward breast cancer cells through ligand-receptor-mediated endocytosis. Conjugating folic acid with chitosan has been shown to increase the cytotoxicity of drug-loaded nanocarriers toward breast cancer cells and their biocompatibility with normal cells, as demonstrated in several studies [69,80,81,82]. Nevertheless, it was clear that the encapsulation of the TO in the CS/Alg-NCs significantly enhanced their cytotoxicity toward the breast cancer cells. Therefore, the use of TO-CS/Alg-NCs can be proposed as a potential alternative therapeutic strategy for breast cancer treatment. Further optimization studies are required to appropriately design the nanoformulation for intravenous administration with minimal off-target effects on normal cells.

## 4. Conclusions

TO-CS/Alg-NCs containing poloxamer 407 as a non-ionic surfactant were prepared using the o/w emulsification, ionotropic gelation, and freeze-drying method with a CS/Alg mass ratio of 0.03:1, 0.65% poloxamer, and 1% TO. The prepared NCs were spherical in shape, with an average particle size of 200 nm and a zeta potential of −21.8. The EE of the TO was 70. The FTIR analysis confirmed the PEC between the primary amines of CS and the carboxylic groups of Alg. The PEC formation was also confirmed by the significant change in the surface charge of the TO-Alg-NCs following CS coating. No new chemical entity formation was observed, indicating chemical compatibility between the TO and the CS/Alg-NCs. The sustained release of TO of approximately 50% after 12 h in both media suggests that CS/Alg-NCs can be used as sustained and controlled nanocarriers for TO. The cytotoxicity of the TO-CS/Alg-NCs was significantly more potent than that of the unencapsulated TO against both the MDA-MB-231 and the MCF-7. With a computed EE of 70%, we can assume that the unencapsulated or unbound TO was 30%. The cytotoxicity of the TO-CS/Alg Alg-NCs was likely to have been due to both the encapsulated and the unencapsulated TO. In the future, the enhanced cytotoxicity of TO-CS/Alg-NCs against cancer cells can be further improved by the functionalization of polymeric materials with cancer-specific ligands for developing active targeted delivery systems.

## Figures and Tables

**Table 1:** Comparison of release kinetics model of TO from CS/Alg-NCs in different media.

Medium	Zero-Order		First-Order		Korsmeyer–Peppas			Hixson–Crowell	
	k_0_	*r* ^2^_0_	k_1_	*r* ^2^_1_	*n*	k_k_	*r* ^2^_k_	k_H_	*r* ^2^_H_
pH 5.5	3.705	−0.5653	0.078	0.4225	0.356	20.650	0.8352	0.018	0.1962
pH 7.4	2.813	0.1404	0.0654	0.6207	0.455	11.442	0.8390	0.013	0.4935

**Table 2:** Mean IC_50_ values of unencapsulated TO and TO-CS/Alg-NCs against MDA-MB-231, MCF-7, and HEK293 cell lines.

Cell Line	IC_50_ (µg/mL)	
	Unencapsulated TO	TO-CS/Alg-NCs
MDA-MB-231	329.53 ± 8.06	99.11 ± 3.40 *
MCF-7	344.60 ± 42.5	82.88 ± 4.40 *^,^^
HEK293	141.33 ± 11.09	84.30 ± 9.60

ANOVA results: * *p* < 0.0001 (compared to unencapsulated TO), ^ *p* = 0.8765, no significant difference (compared to mean IC_50_ of TO-CS/Alg-NCs in MDA-MB-231).

## Data Availability

All the data are available within the manuscript.
